# Strategies to Implement a Community-Based, Longitudinal Cohort Study: The Whole Communities-Whole Health Case Study

**DOI:** 10.2196/60368

**Published:** 2024-12-05

**Authors:** Lindsay Bouchacourt, Sarah Smith, Michael Mackert, Shoaa Almalki, Germine Awad, Amanda Barczyk, Sarah Kate Bearman, Darla Castelli, Frances Champagne, Kaya de Barbaro, Shirene Garcia, Karen Johnson, Kerry Kinney, Karla Lawson, Zoltan Nagy, Laura Quiñones Camacho, Lourdes Rodríguez, David Schnyer, Edison Thomaz, Sean Upshaw, Yan Zhang

**Affiliations:** 1 Center for Health Communication The University of Texas at Austin Austin, TX United States; 2 Office of the Vice President for Research, Scholarship and Creative Endeavors The University of Texas at Austin Austin, TX United States; 3 School of Advertising and Public Relations The University of Texas at Austin Austin, TX United States; 4 Department of Population Health The University of Texas at Austin Austin, TX United States; 5 School of Social and Behavioral Sciences Marymount University Arlington United States; 6 Department of Psychology University of Michigan Ann Arbor, MI United States; 7 Ballmer Institute for Children’s Behavioral Health The University of Oregon Eugene, OR United States; 8 Department of Physical Therapy, Movement, and Rehabilitation Sciences Northeastern University Boston, MA United States; 9 Department of Psychology The University of Texas at Austin Austin, TX United States; 10 Steve Hicks School of Social Work The University of Texas at Austin Austin, TX United States; 11 School of Nursing The University of Texas at Austin Austin, TX United States; 12 Department of Civil, Architectural and Environmental Engineering The University of Texas at Austin Austin, TX United States; 13 Department of Surgery & Perioperative Care The University of Texas at Austin Austin, TX United States; 14 Department of Educational Psychology The University of Texas at Austin Austin, TX United States; 15 David Rockefeller Fund New York City, NY United States; 16 Department of Electrical and Computer Engineering The University of Texas at Austin Austin, TX United States; 17 School of Information The University of Texas at Austin Austin, TX United States

**Keywords:** community-based, longitudinal, health disparities, cohort study, case study, family health, child, children, families, child development, mobile phone

## Abstract

This paper discusses the implementation of the Whole Communities-Whole Health (WCWH) initiative, which is a community-based, longitudinal cohort study. WCWH seeks to better understand the impact of location on family health and child development while also providing support for families participating in the study. Implementing a longitudinal study that is both comprehensive in the data it is collecting and inclusive in the population it is representing is what makes WCWH extremely challenging. This paper highlights the learning process the initiative has gone through to identify effective strategies for implementing this type of research study and work toward building a new model for community-engaged research. Through iterative testing following the Plan-Do-Study-Act model, three main strategies for implementation were identified. These strategies are (1) creating a data collection schedule that balances participant burden and maintains temporality across data types; (2) facilitating multiple opportunities for qualitative and quantitative input from faculty, families, and nonparticipant community members; and (3) establishing an open-door policy for data analysis and interpretation. This paper serves as a guide and provides resources for other researchers wanting to implement a multidisciplinary and community-based cohort study.

## Introduction

Conducting a community-based, longitudinal cohort study with an interdisciplinary university research team is a grand challenge. For the university, the success of the project relies on having a shared vision, collaboration, budget, staff, volunteers, and other support. On the community’s side, the project relies on the community’s motivation to participate, communication, and trust in the research process, particularly when the study focuses on marginalized and underrepresented groups.

This paper discusses the implementation of the Whole Communities-Whole Health (WCWH) initiative, which is a community-based, longitudinal cohort study taking place in Austin, Texas. The initial concept of WCWH emerged from the idea that where a person lives can impact their health, as the study focuses on a community in Austin, Texas, called Del Valle. This community has access to fewer resources and therefore faces more health disparities than surrounding communities in Austin. Research shows that where an individual lives plays a significant role in determining health outcomes (eg, location can determine access to health care, environmental concerns, and proximity to grocery stores with healthy food) [1-3]. As the WCWH initiative continued to develop and grow, it became a project that focused specifically on family health and child development while measuring multiple types of health exposures and outcomes over a long period. This longitudinal effort offers the most comprehensive evaluation of causal relationships between variables. These types of research efforts are additionally strengthened by investing the time and effort to ensure a study is accessible and trusted by families belonging to racial and ethnic minorities, who are too often excluded from health-focused research studies.

WCWH seeks to better understand the impact of physical and emotional adversity, biology, and the environment on family health and child development while also providing support for families participating in the study. The WCWH initiative has many moving parts, and technology plays a key role in the ongoing efforts and success of WCWH. From collecting survey data through a mobile app, tracking physical activity through wearable devices, and using home monitoring systems to measure air quality, technology is present in nearly all aspects of the initiative. Using technology provides an opportunity for researchers to improve their understanding of health behaviors and challenges while also understanding the relationships between measures that affect health over time.

Implementing a longitudinal study that is both comprehensive in the data it is collecting and inclusive in the population it is representing is what makes WCWH extremely challenging. This paper highlights the learning process this initiative has gone through to identify effective strategies for implementing and building a new model for community engaged research. Through iterative testing following the Plan-Do-Study-Act (PDSA) model, 3 main strategies for implementation were identified [4,5]. These strategies are (1) creating a data collection schedule that balances participant burden and maintains temporality across data types; (2) facilitating multiple opportunities for qualitative and quantitative input from faculty, families, and nonparticipant community members; and (3) establishing an *open-door* policy for data analysis and interpretation. This paper serves as a guide and provides resources for other researchers wanting to implement a multidisciplinary and community-based cohort study.

### WCWH Background

#### Overview

The WCWH grand challenge was initiated to change the way science helps society thrive, with a focus on understanding the impact of physical and emotional adversity, biology, and the environment on health outcomes. A large part of achieving this is through the WCWH cohort study, which involves working with families living in the Eastern Crescent of Austin, Texas, over a 5-year period. Between August 2021 and December 2021, the idea became a reality as WCWH worked with the first 15 families (the “ambassador families”) to review and refine the data collection protocols. Within the first year, WCWH built an initial research model to conduct interdisciplinary, community-based research and improve access to research within the community.

WCWH focuses on the health outcomes of families living in or near the Del Valle region of Travis County, Texas, east of Austin. An initial geographic selection process was carried out in the years leading up to the study, which identified rural areas 15 to 20 miles outside of Austin as the most appropriate study area of focus to better understand the impact of location on family health and child development. These residential areas are closely located to an international airport and major highways, which can contribute to an increase in air pollution [6]. Prior work has already shown an increased use of emergency room care for asthma-related illnesses in areas with higher levels of air pollution [6]. A large proportion of residents living in those areas are also families of color, creating racial and ethnic health disparities [6]. In addition to understanding why certain health outcomes differ for families, it is important to identify when outcomes are similar, especially when one group faces barriers or challenges and the other does not. This can help identify protective factors that are linked to family dynamics, relationships, and other social supports that closely tied communities provide [7].

To better understand the complex relationships between multiple health factors, the same families are followed over a 5-year period in the WCWH cohort study. The data collected are returned to families through data return reports, which are distributed throughout the study, giving families the opportunity to view personalized results about their health and their family’s health. How families use this information is up to them, but the goal of giving data back throughout the research study, rather than after the research is published, is a key effort in reaching a better balance in benefits between the researcher and participant. Furthermore, ongoing data feedback creates more transparency regarding what data are being collected and what research questions are being asked.

#### The Social Ecological Model

The Social Ecological Model (SEM) was used when developing the WCWH cohort study, and geographic location was the launching point for WCWH. The SEM is commonly used to understand the interactive effects of personal and environmental factors that determine behaviors and outcomes [8]. This framework helps to identify exposures at the individual and group levels that can impact health over time. It also alludes to the potential interactions between different levels and can better inform prevention practices in the future. Within the WCWH cohort study, the SEM is applied in a community-based and interdisciplinary setting over a 5-year period, which allows for multiple levels to be measured over time versus cross-sectionally.

Cross-sectional studies analyze data at a specific point in time and do not allow causality to be inferred between the different levels of the SEM and the outcome variables. In cross-sectional studies, only a significant association can be inferred [9], which limits the application of the findings. Longitudinal studies provide a more comprehensive research approach and allow researchers to track changes over time.

Most studies using the SEM evaluate only 1 or 2 levels of the model and are cross-sectional [9-12]. It is much less common to evaluate multiple levels within a single study and measure data longitudinally. The WCWH cohort study evaluates a complex variety of variables to understand the impact on health over 5 years, setting it apart from other studies. For example, a more traditional study using SEM and examining child obesity as an outcome would account for the child’s reported eating habits and their physical activity levels (both individual-level exposures). The WCWH cohort study uses multiple SEM levels by looking at eating habits, physical activity levels, family culture and traditions, neighborhood safety, and stress levels of the parent, which extend beyond the individual level and allow us to understand both the relationship between personal factors and health outcomes for the child and how those are impacted by factors external to the child.

#### Study Population

Families are eligible for participation if they have at least 1 child aged ≤4 years and live in or near the Del Valle region of East Austin, Texas. Participation is open to any family or nonfamily member residing in the same household on a full-time basis. As of September 2024, the study has 154 families and 486 participants. Participants are compensated monthly, with annual compensation depending on how many family members are participating: 1 adult and 1 child can earn up to US $815 per year, 2 adults and 1 child can earn up to US $1090 per year, and 2 adults and 2 children can earn up to US $1215 per year.

#### Child Age Criterion

The child age criterion was included because WCWH researchers wanted to test how different exposures impact child development. The age range was selected to primarily capture exposures within critical developmental windows for infants in the first 2 years of life. Capturing health exposure information during critical developmental windows is important for understanding their impact on child development later on. However, because the study includes measures across multiple disciplines, the importance of timing within life stages can differ for each, which is why certain measures, devices, and surveys are for different age groups. Including children between 3 and 4 years old within the inclusion criteria allows for broader recruitment and critical development windows to be examined. This means some children are excluded from measures specific to children between 0 and 36 months. In sum, having a broader age range gives more flexibility for recruitment while still being able to measure exposures during critical windows of development.

#### Recruitment

Participants are primarily recruited through local clinics, childcare centers, schools, community events, partner organizations, Facebook (Meta Platforms, Inc) groups, and word of mouth. Since the start of the recruitment period to present (September 2024), 1140 individuals have been contacted about the study as well as >2000 individuals from using a mass text message service through Women, Infants, and Children (WIC). Of the 1140 individuals contacted through our recruiting efforts, 980 (86%) were lost due to disinterest, loss to follow-up, or ineligibility. While we are not able to consistently collect information regarding why families are not interested, most families comment on being too busy for the monthly time commitments and the length of the study being up to 5 years. There is also sensitivity and distrust around biological samples and mobile app data collection that have served as barriers to participation. Given the scope and duration of the study, having a sample size of 486 participants being followed over time using continuous and direct measures of health still provides meaningful data. Considerations will need to be made for sample bias presented by families who participate, given they may prioritize health and health information more than other families.

#### Del Valle, Texas

Before launching the study, several rural areas of Austin were evaluated based on population size, health care access, food and grocery store access, and demographic distribution. On the basis of these factors, the selected community comprised residents of Del Valle, Texas, and surrounding areas in East Austin, including neighboring counties. Del Valle has been referred to as a “health desert,” with few primary health care clinics and services to meet basic health needs for residents. This lack of health infrastructure results in heavy dependence on emergency care teams and facilities to meet nonurgent health needs. In addition, limited transportation and grocery store access add to the challenges faced by this community. There have been recent pushes from the community for a large grocery store chain to be built in the area [13], as it can be challenging for residents to travel to grocery stores outside of the area, given that public transportation options are limited [13]. In addition, the grocery store options within the Del Valle area have high prices and a limited selection.

#### Enrollment

Within the study, families are enrolled continuously every 2 months. The families enrolled form smaller cohort groups that follow the same data collection schedule. Staggering enrollment like this creates a way for all families to have data collected across all measure types over a 12-month period while giving each of the laboratories and different departments sufficient time to prepare for and later analyze the data being collected.

This study focuses its recruitment efforts in the 5 associated zip codes, including Del Valle, Bastrop, Manor, Caldwell County, Travis County, and Hays County, which are areas served by the Children’s Wellness Center, a partner of the WCWH team. The Children’s Wellness Center was chosen as a partner because they were the only health care provider in the area, had close ties to the Del Valle School District, and were an already-established partner of the University of Texas. This partnership, in addition to other previously established community partners, is used throughout the study period for continued recruitment and engagement purposes to ensure that the study efforts are incorporating community knowledge to inform study questions, aims, and methodologies.

#### Participant Demographics

The study sample is diverse and representative of the Del Valle area. This is due to a majority of recruitment efforts being focused in Del Valle, which has led to the demographic distribution being most reflective of what has previously been reported for the Del Valle Independent School District census data. A major strength of the study is its ability to provide a representative sample population that could be a resource for the Del Valle Independent School District and other community partners in the area. Table 1 shows the demographic breakdown of participants.

**Table 1:** Demographic breakdown of study participants (primary caregiver of the family; N=91).

Characteristics		Participants, n (%)
**Sex of the primary caregiver**		
	Female	87 (96)
	Male	4 (4)
**Race**		
	African American, Black, or African	14 (15)
	Arab, Middle Eastern, or North African	1 (1)
	Asian or Asian American	2 (2)
	Chicano, Latino, or Mexican	36 (40)
	White	23 (25)
**Multiracial**		
	African American, Black, or African; White	1 (1)
	Asian or Asian American; White	1 (1)
	Chicano, Latino, or Mexican; Native American, American Indian, Alaska Native, or Indigenous	1 (1)
	Chicano, Latino, or Mexican; Native American, American Indian, Alaska Native, or Indigenous; Ocean or Pacific Islander	1 (1)
	Chicano, Latino, or Mexican; White	1 (1)
	Choose not to answer	5 (5)
	I do not know	4 (4)
**Education**		
	Less than high school education	7 (8)
	Graduated high school or obtained GED^a^	27 (30)
	Graduated college	20 (22)
	Master’s degree	8 (9)
	Attended some college or trade school	23 (25)
	Professional school degree (MD^b^, DO^c^, DDS^d^, and JD^e^)	3 (3)
	Choose not to answer	3 (3)
**Employment status**		
	Working full time	64 (70)
	Working part time	4 (4)
	Stay-at-home caretaker or homemaker	4 (4)
	Stay-at-home caretaker or homemaker+working full time	1 (1)
	Student; working part time	2 (2)
	Student	1 (1)
	Unable to work; have a disability	2 (2)
	Looking for full-time work	5 (5)
	Not working	7 (8)
	Choose not to answer	1 (1)

^a^GED: General Educational Development.

^b^MD: Doctor of Medicine.

^c^DO: Doctor of Osteopathic Medicine.

^d^DDS: Doctor of Dental Surgery.

^e^JD: Juris Doctor.

#### WCWH Ambassador Families

The first 15 families who were recruited and enrolled in the cohort study are called the “ambassador families.” These families had children aged between 0 and 9 years and lived in or near Del Valle. The ambassador families were the first to go through the data collection protocols and test the new app developed specifically for this study. Throughout this process, ambassador families provided feedback on research components, allowing a smoother process for future participants. Through the ambassador families’ participation, WCWH researchers were able to gauge whether the research methods were nonintrusive and effective in providing insights that connect behavior, attitudes, and environmental factors to health. On the basis of the feedback from the ambassador families, we were able to review and revise the study as needed before scaling up the study’s enrollment.

Ambassador families who had children aged between 0 and 4 years at enrollment could continue in the study after their first year. Families completed all study activities over a 4-month period and completed a follow-up survey and focus group sessions following completion. Families who did not meet the 0- to 4-year age criteria at enrollment as an ambassador family would be able to continue in the study to give feedback on the data reports and have any follow-up testing completed for environmental measures, such as water quality.

### The PDSA Cycle

The use of repetitive testing and adaption cycles emphasized by the PDSA model is a crucial part of creating a model for study implementation that is effective at scale and applicable to multiple settings. Due to the scope of data types being collected, the number of devices being used, and the need for responsiveness to families and their expertise, the PDSA model creates the time and specificity for changes that are a constant requirement for success.

#### PDSA Cycle as a Guide for Research Study Implementation

The PDSA cycle, while more commonly used in the health care setting for intervention and program testing, serves as an effective way for the WCWH study to ground protocols in the main objectives while still allowing for flexibility to respond to participant experience and feedback [14-16]. The WCWH initiative has identified four main research objectives: (1) to examine individual and environmental health determinants involving the expertise of researchers across multiple disciplines, (2) to include a representative sample of families historically excluded from research, (3) to provide opportunities for participants to engage in the research process in an effort to improve the usefulness of the data, and (4) to test new technologies for data collection to increase the capacity of researchers to receive and share data with participants.

These broader objectives are used to guide the planning stage of each PDSA cycle for what is being tested. Simultaneous PDSA cycles are required for each component of the study, including recruitment strategies, data collection, app use, data quality assurance, and data return reports. This paper focuses on the PDSA cycles that were completed after recruitment took place.

#### PDSA Model Used in WCWH

**Figure 1:**
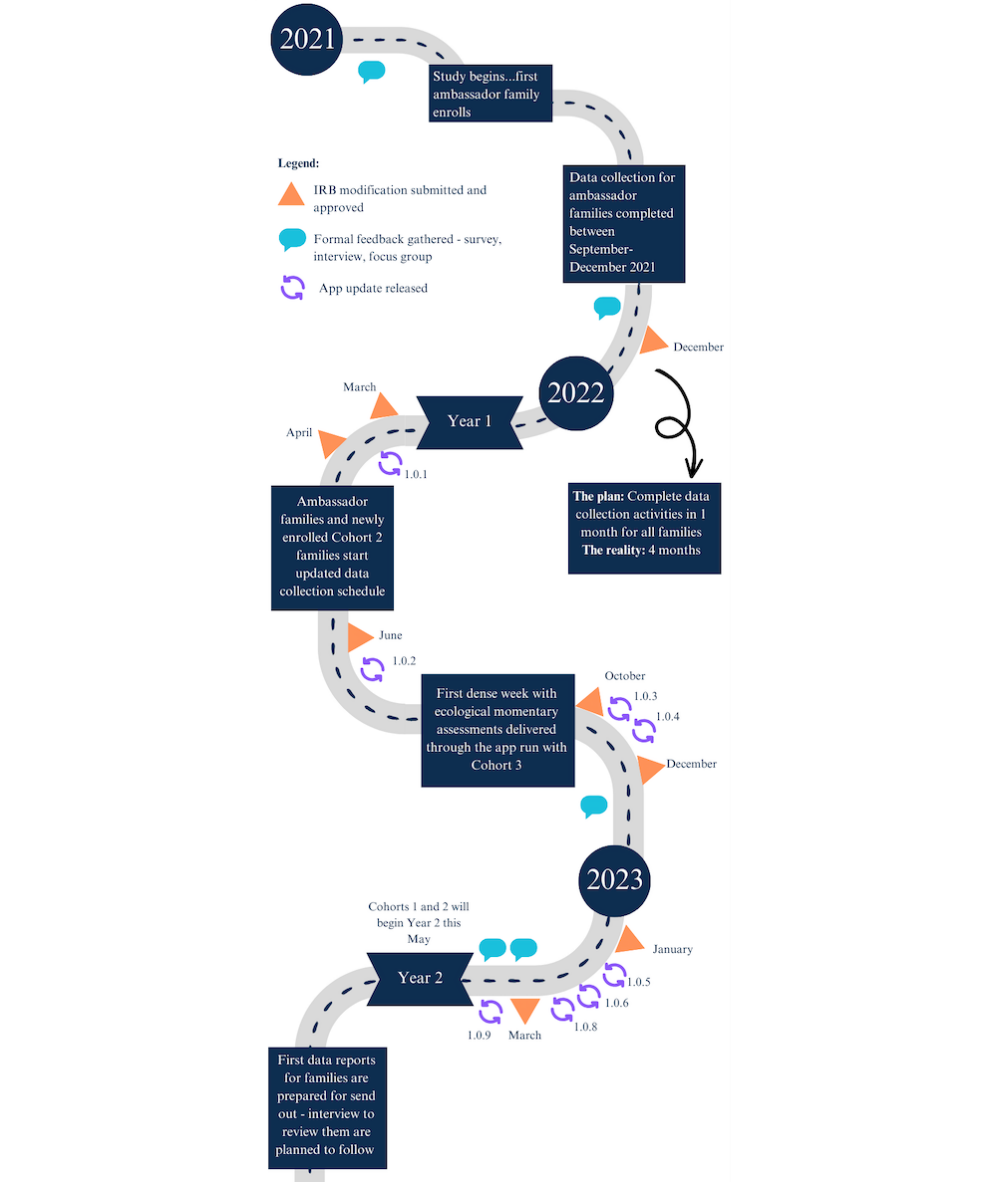
Timeline of implemented changes and formal instances of feedback from families. Changes are grouped into institutional review board (IRB) modification approval and mobile app update release dates.

The first 4 months with ambassador families allowed for PDSA testing with multiple departments and laboratories at the university that work with WCWH. In addition, the community strategy team (CST), who are community leaders in Del Valle, also took part in the testing and revisions for the study during this time and in the months leading up to ambassador family enrollment. The CST members are paid hourly as consultants for the WCWH project and meet monthly to provide oversight and guidance for the study staff and researchers. A timeline for testing, changes, and instances of formal feedback gathering are depicted in Figure 1. This figure combines the simultaneous testing across multiple study components and the changes that followed as a result.

#### Conclusions

Overall, the PDSA cycle acts as a guide for implementing iterative testing, which is imperative to the success of the WCWH initiative. The combined experience of participating families, faculty, and nonparticipating community members was all separate PDSA test groups that led to the development of the 3 key strategies discussed in this paper.

Using an iterative testing model like the PDSA in the context of a research study is both challenging and necessary for improving research conduct and protocol. It was especially important for the WCWH initiative to identify a model of implementation for community-based research. While it does create instances of change that can impact results, if those changes are measured, one then has a way to understand what is working and what is not. For example, we were able to identify when initial changes to our survey review process did not result in significant improvement in survey completion rates. We established a time frame of 4 months to see whether the changes we made were effective or not, defined what a successful outcome would be (90% completion), and then at the 4-month review point, when we did not see the defined successful outcome, we knew we needed to shift and try something else. This model allows for quick indicators of success and failure and incorporates scheduled times of pause for review, all of which have guided the WCWH team to create their initial model for implementation.

### Strategy 1: Data Collection

#### Overview

The first strategy in implementing WCWH focuses on creating a data collection schedule that balances participant burden and maintains temporality across data types. Having a strategy for data collection is imperative in allowing sustainable research and maintaining a positive and trusting relationship between researchers and the participating families. This was one of the first challenges discovered through the interaction with ambassador families. All data collection for the group of 15 families was proposed to take place over a 4-week period. In reality, this process took 4 months for all families to complete data collection activities. Faculty also faced quick turnaround times to have supplies prepped to meet the limited availability families had for scheduling. As sample collection and device use were ongoing, participants were also given over 29 surveys to complete. Overall completion was relatively low across study activities and led to the re-evaluation of the initially proposed study timeline.

The first goal was to reduce participant burden. Participants now take smaller survey sets 4 times a year, with the surveys being limited to 1 to 1.5 hours in length per set. For data collection requiring high-cost devices (such as those measuring air quality) and longer preparation times, data collection is staggered to allow for equipment rotation and more time for kit preparation. In addition, there are gaps in this type of data collection to allow for laboratory groups to review their data and troubleshoot for the next round of data collection.

Participants are followed over a 5-year timeline, on a 12-month calendar schedule; meaning, measures are collected and repeated each year during the same months. This allows researchers to better test differences over time while not burdening participants to complete multiple study activities all at once. During the 12-month calendar, there is a 4-month window with minimal study activities, allowing families a break after the more intensive months. Spreading out data collection in this way helps resolve some of the burden and capacity issues faced by ambassador families but also creates additional challenges in maintaining temporality across multiple data types.

Considerations for multiple data types and participant burden were crucial to creating the data collection schedule. The timing of measures was based on which relationships were most dependent on the timing overlapping with other measures, and if there was flexibility, it would be moved to the next month. For example, it was very important to ensure that child behavior surveys were delivered the same day as a child wearing the audio recording device or that dust samples and the air sensor placement coincided. Coordination is necessary for capturing reliable information and establishing relationships in the long term. The data collection calendar, which shows a breakdown of each month’s collected measures, is shown in the Multimedia Appendix 1. Multimedia Appendices 2-5 show focus group guides (English and Spanish), a list of all measures collected, and survey materials, respectively.

To account for conditions susceptible to change, such as air, water, and smells, there is optional reporting through the mobile app that is available for participants to use and submit at any time. These reports are time and location stamped and would allow for identification of community-level issues as well. Related measures (eg, dust and air quality, physical activity and sleep, and child behavior and language environment) are strategically measured simultaneously to test causal relationships. Along with the surveys, ecological momentary assessments (EMAs) are sent to participants every 2 weeks, which play an important role in collecting data on key study constructs that relate to the other devices and sample data being collected during different months. The EMA asks a participant to respond to questions about the past 2 weeks and covers key study constructs, including social support, caregiver stress, anxiety, depression, and experiences of discrimination.

#### Technology Used for Data Collection

For data collection, WCWH uses different types of technology to collect a wide variety of environmental (eg, drinking water quality), behavioral (eg, physical activity), biological (eg, cortisol levels), and psychological (eg, mood) variables. By collecting such a wide array of measures and health determinants, researchers are able to see a more holistic picture of health over time.

#### Mobile App: Hornsense

One of the main data collection features of the WCWH initiative is the Hornsense mobile app that allows for continuous data collection over time. The mobile app was developed both with the goal of providing additional real-time data collection and increasing accessibility to research study participation. The app creates a way for all families to complete surveys from their homes and do them at a convenient time in their schedule. All participating families must download the Hornsense app, and it is available on both iOS and Android. The app is the home base for study information. It is used for survey completion, sending EMAs, and presenting collected data back to participants. The app was developed over several years, using both a student sample and the ambassador families for testing. Using the ambassador families for testing the app helped the WCWH research team refine their methods before scaling up to the full set of participants.

Participating families receive EMAs and surveys through the Hornsense app. The survey measures vary depending on the month. These surveys are relatively short in length and take about 1 to 1.5 hours to fill out. Limiting the survey length eases participant burden, with the goal of having participants be more likely to fill out all the surveys each month. In addition, the surveys do not need to be completed in 1 sitting; participants can pace out their surveys throughout the month.

The Hornsense app is also able to passively collect GPS, motion, pollen, weather, and mobile device data. Integrated sensors on smartphones provide access to information that tells how participants interact with the app, their mobile device, and how they move within the community. The app is integrated with wearable and sensing devices whose data are used to study the personal and public health of the community.

One of the shortcomings of long-term research projects is that study results are not shared until the end of a study, when they may no longer be useful or have the same impact. To meet this challenge, the Hornsense app serves as a mobile dashboard that translates data collected during the study into actionable, real-time information, which is accessible to interested study participants and community groups.

#### Overview of EMAs

EMAs are an important feature of the Hornsense app and data collection. Through the app, participants are delivered EMAs every 2 weeks to co-occur with all measures. In addition, there are 2 dense data collection periods where EMAs are sent daily for 1 week. These are specific to the devices being used during those periods, including the Language Environment Analysis (LENA), accelerometer, and Fitbit.

The Hornsense app allows for EMAs to be delivered at specified times as surveys, and each is only available for a specific period ranging from 4 to 24 hours, depending on the type of assessment being measured. In previous research, EMAs as SMS text messages have been used to measure a wide variety of in-the-moment variables, such as mood [17], mental health [18], and substance use [19,20] and have been proven useful in collecting in-the-moment measures [21]. For WCWH, the EMAs measure parenting stress, sleep, mood, mental health, physical activity, discrimination, and social support.

#### Activity and Sleep Monitors

In addition to the mobile app, WCWH relies on an array of technological devices to collect data. Over a 12-month period, adult participants wear Fitbit watches (Google LLC) for 2-week periods, which occur 4 times a year. In addition, participants have the option to continue to wear the devices outside these designated data collection periods. The Fitbit watches measure both movement and heart rate (resting and active). From those signals, daily sleep and physical activity parameters are determined. With parental consent, children aged ≥3 years wear an accelerometer (activity monitor) for up to 7 days every 6 months or twice a year.

#### LENA Technology

The LENA device is a lightweight audio recorder that fits in the pocket of a vest worn by a toddler. It is designed to measure conversational turns for children aged between 0 and 36 months. A conversational turn is a back-and-forth communication interaction between a child and an adult, and it occurs when a child speaks and an adult interacts, or vice versa, with no more than 5 seconds in between [22]. The device captures any words or noise an infant or toddler makes and any other conversation within 1.83 to 3 meters of the child wearing the device. Previous LENA research has indicated that conversational turns play a role in early brain development. A higher number of conversational turns has been shown to increase reading skills, vocabulary skills, social emotional development, and reasoning scores [22].

#### Home Environment Monitors

In the first month of the study, participants receive a home air sensor, which measures temperature, humidity, carbon dioxide, and ambient noise levels [23]. These items are important to measure, as air pollution has a direct effect on health and pediatric respiratory conditions [24]. In addition, noise pollution, which is noise around the home, such as construction or traffic, negatively impacts sleep and mental health, which impacts overall health and well-being [25].

#### Conclusions

Each area of the study described earlier requires testing and feedback loops to ensure that data collection is consistent, reliable, and complete. The changes made to the data collection schedule allowed the study team to fulfill data collection needs, not overburden participants or study staff and faculty, and more consistently collect data over time across health measures. In addition, the use of multiple devices measuring a wide variety of health measures (eg, environmental, behavioral, biological, and psychological) provides a more holistic picture of health over time.

When collecting data, the burden on participants is kept in mind, as the data collection is flexible and spaced out over a year. A systematic literature review on retention strategies in longitudinal cohort studies determined that one of the most effective strategies in cohort retention is reducing participant burden (such as providing flexible data collection methods) [26]. The overall retention rate of the WCWH cohort study has been very high, with an average of 92% across cohorts who have participated for 2 years and an average of 87% across cohorts who have participated for 1 year. Only 1 (0.6%) of the 154 families withdrew from the study due to the feeling that they would not have enough time to participate in all activities, and 13 (8%) of the families were lost to follow-up, which is the status designated after 10 contact attempts over 3 months with no response. We believe one of the reasons for the high retention rate for the cohort study is the flexibility of the study and data collection as well as staff efforts and participants’ intrinsic motivation for participating in the study.

Retention strategies are not often documented within study protocols, although there are some key strategies WCWH has followed that are cited by longitudinal studies with high retention rates [27]. These include collecting multiple forms of contact information, providing financial incentives, building community trust, partnering with health care centers, individualizing retention strategies for participants, and having culturally competent staff [27-34]. Due to the multiple backgrounds and experiences represented by the families participating, it has been crucial for WCWH to tailor retention strategies to each family.

WCWH staff play a large role in the high retention rate of the study. Staff prioritize family needs, experiences, and expertise. When participating families are initially enrolled, WCWH staff first address the basic needs of families before asking for additional data. Immediate needs are addressed during the first home visit (eg, food, clothing, and shelter), and following this visit, resource lists with additional information are provided. Staff also offer points of engagement outside of scheduled data collection activities. Participants are able to engage with staff during WCWH-related community events. These events are outside of data collection and the study; however, they provide information and resources that have been useful to participating families and the CST. These events are not directly tied to study goals or meeting the research objectives; however, they build trust and partnership between WCWH and the community, which is a core aspect of the WCWH model.

In addition to staff efforts, participants are intrinsically motivated to continue participating in the study. In the focus groups with ambassador families, participants were asked about their motivations for participating in the study. Participants stated they continue taking part in the study due to interest in learning more about their family’s health and curiosity in learning new things that are not typically readily accessible, and these 2 factors can contribute to the data return reports. Furthermore, participants mentioned feeling satisfaction in being part of an initiative they view as meaningful and impactful.

### Strategy 2: Team Input

The second strategy used to implement the WCWH initiative is facilitating multiple opportunities for input from faculty, community members, and families. WCWH is a collaborative effort, and the success of WCWH is largely due to input and feedback from multiple sources.

#### The WCWH Team

The WCWH team includes staff, researchers, student researchers, volunteers, community liaisons, community advisors in the CST, and faculty across a university campus. The faculty involved in the project conduct research and steer the goals of the WCWH initiative. Faculty come from over a dozen departments on campus, including engineering, psychology and psychiatry, advertising and public relations, nursing, kinesiology and health education, and population health. Each faculty member brings in their expertise and advises on the research conducted for WCWH.

One of the most important aspects of the WCWH initiative is the CST, which consists of community advisors and liaisons, most of whom are members of the Del Valle community. CST members provide valuable feedback on how WCWH can engage the community in research, with the goal of making the research accessible and relevant to the community. The CST meets monthly to help review ongoing study activities and data reports, as well as to help revise study messaging and public-facing materials. In addition to these efforts, the CST also coordinates workshops and events for the community that are open to families and community members in the WCWH study area. This provides the initiative with a mechanism to contribute to the community and provide resources and information in an effort to acknowledge the current issues or topics that families want to know about. For example, during the study period, there was a vote at Austin City Council regarding the construction of jet fuel tanks for the airport in close proximity to residential areas in Del Valle. The CST planned an event with both environmental experts from the university and a local organization, People Organized in Defense of Earth and Her Resources, to hold a discussion on the plans for construction and how this might impact their neighborhood and health. These types of events and discussions help the overall WCWH initiative stay true to its core objective by cultivating meaningful partnerships between researchers and community expertise while also being aware of current events that impact the people in the community. More importantly, by having this type of event organized and led by the people from the community and supported by the university, it creates a tangible way for sustaining relationships in the area that go beyond the research itself.

Previous research demonstrates the advantage of having community input through community advisory boards [35-37]. Through the WCWH CST and community liaisons, the project is able to get input from individuals who are in the community under study. Like a traditional community advisory board, the WCWH community team broadly oversees how the research being conducted is practical and applied; however, the members of the CST and the community liaisons are also involved in the development and growth of the initiative. The community team is involved in each stage of the process (eg, workgroups, community events, and recruitment) to ensure the voices and concerns of the Del Valle community are heard. When conducting research with a community that has been historically marginalized or underrepresented, building and maintaining trust is imperative, as mistrust of researchers exists due to past injustices [38].

#### Team Input and Community Feedback

Interdisciplinary research is not a new concept, and there have been increasing shifts among all research institutions to promote this approach in health research [39]. However, there is a gap in how to best conduct this type of collaboration and combine it with community-engaged research. Input and feedback from faculty and families produce a collaborative environment. Therefore, creating time and space for these conversations has been an important goal for the WCWH study throughout. Faculty involved with the initiative receive at least biweekly updates about the project as well as monthly steering committee meetings where all faculty get together to discuss research, the current status of WCWH, and future goals for the project. Along with the steering committee meetings, faculty attend the different workgroups that occur biweekly. The workgroups focus on a range of topics, including recruitment and retention, data analysis and interpretation, and data return report development. Community liaisons, many of whom are also CST members, attend all the workgroups alongside faculty, providing input and expertise in these areas.

Community liaisons in the data return workgroup provide invaluable feedback on how to structure the reports with results for participants, what type of verbiage should be used, how much text and details to include, and more. Once these reports are developed and finalized by the team, they are sent to participants. For the first few reports, in-depth interviews were conducted with participants to get direct feedback on these reports, and adjustments were made for future reports.

Another feature that heavily relies on faculty, CST, and family feedback is the Hornsense mobile app. The WCWH faculty and CST members have app test accounts where they can download the app on their phones and test the app for bugs or other malfunctions. Families using the app can report any app issues they have through the app or a feedback form. In addition, feedback surveys are given to participants to share their thoughts on the level of difficulty of study tasks, data interest, and device use.

#### Ambassador Family Focus Groups

To receive participating families’ feedback and input, WCWH researchers facilitated focus groups with the ambassador families to assess their experiences with the longitudinal study. The aim was to gather insights into the families’ understanding of the study’s goals, their motivations for their initial and continued involvement, their experience with the tasks required to complete the study, and their experience in providing and receiving feedback. Two focus groups were conducted—1 in English and 1 in Spanish. The main findings of these 2 focus groups are summarized.

#### Understanding the Study’s Goals

The study was perceived as playing a crucial role in uniting the community toward improved living conditions, with an emphasis on its potential contribution to community development and empowerment. Spanish-speaking participants highlighted the educational and resource-oriented aspects of the study, such as receiving information about English classes and food distribution. The study’s focus on environmental health, including water and air quality, resonated well with the community’s interests, contributing to a holistic understanding of well-being.

#### Motivation to Participate in the Study

Motivations for participating in the study were varied. Initial curiosity was a starting point, often prompted by engaging recruitment strategies, such as offering free books at community events. Participants were drawn to the study by the promise of better understanding their diverse community and contributing to collective well-being. The desire for personal and community development was a common theme, with participants expressing motivation to learn new things not readily accessible outside the study’s framework.

Monetary compensation, in the form of gift cards, varied in its impact on motivation. While some acknowledged it as a motivating factor, others viewed it as secondary compared to the satisfaction of being part of and contributing to a meaningful initiative. Participants seemed to be mostly altruistically motivated, having a desire to contribute to the larger good of their community and witness improvements in their neighborhood.

Participants’ commitment to stay involved in the study became a personal value for some, driven by a sense of duty or responsibility. However, this commitment also led to feelings of guilt for those unable to maintain their initial level of engagement due to challenges, such as limited time.

#### Participants’ Experience With the Study Design

Participants provided feedback on their experience with the study design. Participants encountered difficulties with several technical aspects of the study, particularly the Hornsense app and accelerometer device, ultimately impacting their engagement and overall experience. For the app, issues included malfunctioning links, surveys not being saved, and log-in complications. Continued iteration of the app has been essential to improving the user experience for study participants. For the accelerometer, issues included improper fit and discomfort for children. Participants suggested alternative, user-friendly designs, such as elastic materials or widely accepted formats like fitness trackers.

Participants also gave feedback on the time commitment of the study and noted that the frequency and time needed to complete study tasks varied. Some surveys and measures fit seamlessly into participants’ routines, while others presented logistical challenges. The dust and saliva samples were highlighted as quick and straightforward, but the water collection was noted for its time sensitivity, as it required coordination with pickup schedules. However, participants appreciated the study’s flexibility, noting that the research team was accommodating when scheduling issues occurred. Despite challenges, participants found the study’s requirements generally reasonable and feasible, suggesting potential improvements win task design and scheduling adjustments.

#### Focus Group Discussion

Participants’ feedback and reflections on their involvement in the study highlighted the complexities of conducting research in diverse communities, along with the importance of communication, trust, and the practical use of technology. Participants praised the empathy and responsiveness of the study coordinators, who were commended for their attentiveness and problem-solving approach. The personal approach, with home visits and direct contact with the research team, appeared to foster trust and improve communication, as it added a layer of authenticity and seriousness to the study. However, technical difficulties, particularly with the study app, caused frustration. Participants suggested a more rigorous testing phase for the app on various devices to ensure functionality.

Moreover, participants stressed the importance of transparency and communication when it came to the use of personal information. One of the participants expressed concerns about the potential misuse of sensitive data, such as DNA from biological samples. The participant shared that there should be clear communication about its security and use. It is important to note that providing biological samples is optional to participate in the WCWH cohort study.

For incentives, participants preferred a more straightforward form of compensation, such as prepaid Visa cards. They reported that the process of redeeming gift cards was cumbersome and restrictive, suggesting that a more flexible and accessible reward system would be beneficial, especially for those who might rely on these incentives for essential needs.

Overall, the study had a positive influence on the participants’ perceptions of research, reinforcing their desire to contribute to change and community improvement. The sentiment was that university-led research felt more trustworthy. Ensuring that technological tools are user-friendly, respecting participants’ privacy, and providing flexible compensation can enhance the experience and maintain engagement in community-based research. The reflections also indicated that personal interactions and responsiveness from research teams can build trust and contribute to participants’ positive perceptions of their involvement in research initiatives.

### Strategy 3: Open-Door Data Policy

The third strategy for the implementation of WCWH is establishing an “open-door” policy for data analysis and interpretation. This policy includes creating transparency and frequent exchange about data collection, data storage, and data use with families and researchers. Emphasizing the ownership families have over their data has been a key effort for this study. Some of the specific efforts made to create a stronger connection between researchers and participants that help reach a better balance of both benefit and power when it comes to data ownership and use are mentioned subsequently.

The data and measures collected for WCWH exist to benefit the participants. The first way WCWH set out to ensure data being collected were guided by participant interest was by requiring only two things for active study participation: (1) downloading and using the study app and (2) completing surveys. All other study activities and measures (eg, Fitbit watches, medical record access, and biological samples) are optional, and participants can choose which activities they wish to participate in. Setting up this expectation at the beginning of the study creates a more transparent relationship between faculty and participating families.

Throughout the study, the research team emphasizes where the data go, how the data are stored, and what the data are used for. Participants have multiple ways to access their data through viewing it in the app or through the data reports. Data accessibility includes their ability to request data being deleted at any point in the study. The data collection and storage process is meant to be completely transparent with the participants. In addition, the survey instruments used to collect data include citations for participant reference. Families can also ask why measures are worded in specific ways, and the researchers can explain how the survey instrument was developed and validated.

The data return reports play a large role in data transparency for the participating families. The data collected for WCWH is returned to participants in the form of data return reports periodically throughout the study. These reports are designed by researchers, and feedback is initially provided by a team consisting of community advisors, staff, faculty, and researchers. After a final draft is curated, focus groups with participating families provide feedback on the reports. These reports transform the complex data that are collected into easily understandable information for participants, with additional resources provided if the report results are out of the normal range. The data return reports allow participants to take immediate action to improve their health, as opposed to years later when the research is published in an academic journal.

## Discussion

The WCWH initiative is constantly developing and evolving to determine best practices, and the use of technology is integral to the study. As more families are recruited and the study grows, the WCWH team will be faced with new challenges. Setting up testing cycles using the PDSA model will continue to be important for the successful implementation of this initiative.

The WCWH initiative is unique in two very significant ways: (1) participants come from historically excluded demographics and (2) the data collected in the study are returned to the participants. These unique factors also create unique challenges, which the WCWH study has used the PDSA cycle to help identify and address.

The strategies described in this paper were identified through testing protocols that were set to meet the overall study objectives. As with any implementation strategy, once it is put in place, unexpected events will necessitate change. The PDSA allows those events to serve as additional data points to evaluate the best option for a change and the subsequent evaluation of whether that change resulted in an improved outcome. This was followed within each aspect of the study. The mobile app has undergone 14 updates since its initial release to respond to study needs and family experiences. The institutional review board research protocol has undergone 18 modifications since its initial approval to also respond to the unexpected events that arose. While some might see these number of changes to a research study as cause for concern, when changes are both defined and measured over time and applied within a validated implementation model like the PDSA, instead of creating data inconsistencies, the most important research question that the WCWH initiative set out to answer is answered: How does a research team conduct a longitudinal, interdisciplinary, community-engaged study in a way that can be sustained and replicated by others over time?

The first strategy focused on data collection. Because of the first main testing cycle with ambassador families, we learned we needed a major revision to the data collection schedule, given the feedback from ambassador families, faculty, CST members, and staff addressing the challenges that arose within the first round of data collection. With the objective of complete, consistent, and high-quality data, we needed to have a schedule that could best fit family schedules and faculty schedules. Otherwise, consistent participation over time would be good for some but not all participants. Likewise, having consistent engagement for faculty members also required making the data supply requests and timing more feasible.

The second strategy focused on team input and feedback. Having a plan for implementation means preparing for success but also preparing to respond when things do not go as expected. Much of what was initially proposed for the study had to be adjusted. Understanding the need for community and participant feedback was crucial for these adjustments. If adjustments were made to increase compliance solely based on faculty feedback without considering the experience of the participating families, then changes made are likely to fail or fall short. In the focus groups held with the ambassador families, their comments related to the survey language, app issues, compensation issues, and accelerometer belt discomfort led to modifications in the protocol. It is important to note that creating opportunities for input means creating a process for response as well. Moreover, a response can mean being honest and transparent when you are not able to respond to certain feedback that requires more time, staffing, and funding, which a research team may not have. Creating opportunities for input also means engaging in partner events, supporting local issues, and providing resources in response to community-identified needs. The CST has played an integral role in making that possible.

The last strategy focused on data transparency. Data transparency has been a guiding principle for the project as a whole. Technology is used for real-time data collection and data return to give greater access and benefits to research participants. Having a PDSA cycle in place when it came to mobile app testing, institutional review board consent language, and data report messaging and resources allowed WCWH to make meaningful revisions and materials for creating and maintaining data transparency during the study. These efforts continue as data reports are created and returned to families.

While these strategies have been successful so far in the implementation of the WCWH cohort study, it is important to note their limitations. Creating a 12-month data collection schedule and a 2-month rolling recruitment window allowed for reduced participant burden monthly. However, it also created a tight schedule for the internal staff team to follow for each cohort, given all data collection visits needed to be completed for all participants within 1 month; this would grow increasingly difficult as participant numbers increased. With a 2-month recruitment window, this also meant each cohort was following a different schedule, which requires intensive tracking and record management. The second strategy for gathering feedback worked well to provide revisions to the study protocols but was also limited in that we would miss out on feedback from less engaged and nonresponsive participants and faculty. Formal feedback opportunities for participants were mainly focused on the ambassador families, who may be more invested in the study or their health compared to other participants who joined in the subsequent years. This could mean the strategies that were developed work well for some but not all families. Finally, data transparency has helped create a more trusting environment between researchers and participants, but the team has had trouble creating and returning data reports in a timely manner to participants. At the current staff and student volunteer capacity as well as the unique requirements for processing different data types, the turnaround time cannot be shortened. In addition, the app has undergone several updates in the past 2 years, and not all participants routinely update their apps. This has led to some having access to additional information and functionalities and others not.

Conducting longitudinal studies has always been a challenge. Conducting a comprehensive and inclusive longitudinal research study presents a grand challenge. Using strategies that both meet the rigor required for respected research and establish trust with the families by acknowledging and respecting the expertise they have in their own lives and that of their children’s lives has resulted in the ongoing success of this study, both in terms of the data being collected and in how we inform future studies who wish to do the same.

## Appendix

Multimedia Appendix 1 Data collection schedule with measures.

Multimedia Appendix 2 Ambassador family focus group protocol in English.

Multimedia Appendix 3 Ambassador family focus group protocol in Spanish.

Multimedia Appendix 4 Data measure table.

Multimedia Appendix 5 Ambassador family feedback survey.

## Abbreviations

CST: community strategy team
EMA: ecological momentary assessment
LENA: Language Environment Analysis
PDSA: Plan-Do-Study-Act
SEM: Social Ecological Model
WCWH: Whole Communities-Whole Health
WIC: Women, Infants, and Children
