# Supplementary material for: Strategies to Implement a Community-Based, Longitudinal Cohort Study: The Whole Communities-Whole Health Case Study
Source: JMIR Form Res. 2024 Dec 5;8:e60368. doi: 10.2196/60368 (PMC11659690; doi:10.2196/60368)
Supplement: Multimedia Appendix 2 [file formative_v8i1e60368_app2.docx]

**
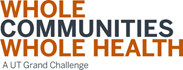
**

**Family Feedback Focus Group Protocol - Fall 2022**

**TOTAL TIME: 1 Hour**

1. **Welcome, Consent, & Ground Rules (10 min.)**
   1. **Welcome people as they arrive**

[*Welcome people, Check in on whether everyone knows the Zoom controls. Support facilitator sends chat msg (#1) to welcome anyone who comes in late.*]

- 1. **Introduction**
     1. Thank you so much for joining us this {evening}.
     2. Name & Title
     3. As you know, Whole Communities–Whole Health is a community-centered research initiative from The University of Texas at Austin committed to the health of children, families, and communities in Austin.
     4. Zoom Check-in/Overview:
        1. Please keep your camera on unless you need to take a break. When you come back, please turn it back on.
        2. Ask if everyone is familiar with muting/unmuting, review if needed
  2. **Purpose/Consent Reminder: READ WORD FOR WORD**

The purpose of this focus group is to hear from you about your experience of participating in the Whole Communities Whole Health research study for the past year. We are interested in learning more about what you think of the study’s goals, the time commitment, and the tasks involved. We will be audio recording this focus group zoom session. We will not identify you in these transcripts or in any publication of the information.

This zoom call should last approximately 1hour. If at any time you decide you no longer wish to participate, you may feel free to leave. If you leave it will not impact in any way your relationship with us or UT.

At the end of our time today, we’ll record your attendance and send you a $25 gift card for your time.

What questions do you have before we begin? OK, we will begin recording now.

- 1. **Group Agreements**

To help our conversation flow, we’d like to establish some ground rules for the group. Group Agreements are meant to help create a safe space where everyone feels comfortable speaking and sharing their ideas. We ask that you follow these, and if you have anything you’d like to add to the list, you are welcome to!

- - 1. Ask that every participant will not take anything said during the focus group outside of this space. (By this we mean what the participants share in the group, not the information on Whole Communities-Whole Health.)
    2. There are no right or wrong answers
    3. All perspectives are respected
    4. Ask for clarification when necessary
    5. Be kind to yourself and respond to your personal needs during the session. (If you have children in the house, we understand that you may need to care for them.)

Does anyone have anything they’d like to add to this list? Can everyone agree to follow these? If at any time it seems that we need to, I will call our attention back to the ground rules.

- 1. **Group Introductions**

**To start our discussion,** I’d like to invite everyone to do a round of introductions, and to share one word that describe your experience with Whole-Communities Whole-Health study. [*Call on someone*], would you like to go first?

1. **Understanding and Motivation (10 min.)**

As an ambassador family, your participation in the initial phase of this long term study has been quite insightful for us here at Whole Communities – Whole Health. Your feedback in this session will help us improve our study design to make sure it is beneficial and useful to other participating families. Remember, you can use the chat to agree with others or answer the questions.

- 1. To start our discussion, I first want to ask you about what you think this study is trying to achieve? (Goals)
  2. What made you want to participate in a study like this?
  3. Why did you choose to continue?

1. **Experience (15 min.)**
   1. What is one task that was the **most difficult** to complete? What aspect made it difficult? Why? What would make it easier?
   2. What do you think of **the frequency (schedule)** of data collection tasks?
   3. What do you think of **the time commitment** for each of the tasks?
   4. Optional probe: As an ambassador family you experienced two different data collection schedules, when you first started everything was done over the course of a few months after you enrolled. Starting the next year we brought a study binder with the new schedule. Having done both – did anything improve or were there changes that made it more difficult?
   5. After participating in this study, would you be interested in participating in future studies with us or other organizations? Why? Why not?
   6. How do you feel about research organizations like universities and government institutions? (Compare before and after participation in the study, what changed?)
   7. What would make you trust a research organization and participate in research studies like this one?
2. **Feedback (10 min.)**
   1. You were asked to give feedback into how the study was conducted, do you feel your feedback was responded to\implemented?
   2. How would you like to receive information about the results of this study? (via phone, in person, Zoom call, written format via email)
3. **Learning Moments (10 min.)**
   1. What did you learn about your family by taking part in this research?
   2. What part of the data collection was the most helpful\interesting to you?
   3. Optional probe: As the study has progressed you have been able to see the Hornsense app from the beginning. As you have started using it more what are things that you think are working and which things still need some improvement.
4. **Reflection: Other questions, ideas, and stories from participants (5 min)**
   1. What questions or feedback that have not been brought up that you would like to add?
5. **Conclusion** **(5 min)**
   1. Thank you for taking the time to share your thoughts with us today. We will use your feedback to improve our study design and ensure that our study goals align with your community’s needs. We are currently working on designing a platform to share back the information that we gathered throughout this study. We will be conducting a round of interviews in January where we meet individually with each ambassador family to get your feedback on the design process for this. A team member will be reaching out to you soon to schedule this interview.
   2. I also want to take this time to bring your attention to a detailed feedback survey that was shared with you earlier last month. I know that some families were not able to access the survey or didn’t receive the survey at all. The problem is fixed now, and you should be able to log in to the study app and complete the survey. It only takes 5 minutes, and it would be very helpful for us to learn about how you evaluate each of the tasks you completed for this study. You will receive a $5 compensation upon completing the survey.
   3. You will receive your gift card by tomorrow (or Monday, if focus group is on a Friday. It will be emailed to you.
   4. Thank you!
