# Supplementary material for: Strategies to Implement a Community-Based, Longitudinal Cohort Study: The Whole Communities-Whole Health Case Study
Source: JMIR Form Res. 2024 Dec 5;8:e60368. doi: 10.2196/60368 (PMC11659690; doi:10.2196/60368)
Supplement: Multimedia Appendix 3 [file formative_v8i1e60368_app3.docx]

**
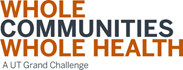
**

**Family Feedback Focus Group Protocol - SPANISH**

**TIEMPO TOTAL: 1 hora**

1. **Bienvenida, consentimiento y reglas básicas (6:00 pm - 10 min.)**
   1. **Bienvenida a las personas a cómo van llegando**

[*Welcome people, Check in on whether everyone knows the Zoom controls. Support facilitator sends chat msg (#1) to welcome anyone who comes in late.*]

- 1. **Introducción**
     1. Muchas gracias por acompañarnos esta {noche}.
     2. Nombre y título
     3. Como saben, Nuestra Comunidad-Nuestra Salud es una iniciativa de investigación centrada en la comunidad por parte de la Universidad de Texas en Austin, con enfoque en la salud de los niños, las familias y las comunidades en Austin.
     4. Zoom check-in
        1. Por favor, mantenga su cámara prendida al menos que necesite tomar un descanso. Cuando regrese, préndala de nuevo.
        2. Pregunten si todos están familiarizados con el micrófono y como silenciar / reactivarlo, repasen la información si es necesario
  2. **Propósito/consentimiento:** **READ WORD FOR WORD**

El propósito del grupo focal es para escuchar sobre sus experiencias al participar en el estudio de investigación Nuestra Comunidad Nuestra Salud durante el año pasado. Estamos interesados en aprender más sobre lo que piensan de los objetivos del estudio, el compromiso de tiempo y las tareas involucradas. Vamos a grabar el audio de Zoom de esta sesión del grupo focal. No se les identificara en estas transcripciones o en ninguna publicación de la información.

Esta llamada de zoom durara aproximadamente 1 hora. Si en algún momento decide que ya no desea participar, siéntase libre de irse. Si se va prematuramente, no le afectará de ninguna manera su relación con nosotros o la universidad.

Al final de nuestro tiempo hoy, tomaremos nota de su asistencia y le enviaremos una tarjeta de regalo de $25 por su tiempo.

¿Alguna pregunta antes de comenzar? OK, comenzaremos la grabación.

**Group agreements – Acuerdos del grupo**

Para asistir con nuestra conversación, nos gustaría establecer algunos acuerdos básicos para el grupo. Los acuerdos del grupo nos ayudan a crear un espacio seguro donde todos se sientan cómodos hablando y compartiendo sobre sus ideas. Les pedimos que sigan los acuerdos, y si tiene algo que les gustaría agregar a la lista, ¡son bienvenidos!

- - 1. Pedimos que nadie divulgue la información que se comparta durante el grupo focal. (nos referimos a la información que los participantes comparten durante este grupo, no a la información sobre Nuestra Comunidad Nuestra Salud).
    2. No hay respuestas correctas o incorrectas
    3. Todas las perspectivas serán respetadas
    4. Pidan aclaración cuando sea necesario
    5. Sean amables con ustedes mismos y respondan a sus necesidades personales durante la sesión. (Si tiene hijos en la casa, entendemos que habrá momentos en que necesiten ponerles atención).

¿Alguien tiene algo que les gustaría agregar a esta lista? ¿Están todos de acuerdo en seguir los acuerdos? Si en algún momento parece que es necesario, llamaré nuestra atención a los acuerdos del grupo.

- 1. **Group introductions**

**Para comenzar nuestra discusión,** me gustaría invitarles a que tomen un turno para introducirse y compartir una palabra que describa su experiencia con el estudio Nuestra Comunidad-Nuestra Salud. [*Llama a alguien*], ¿le gustaría ir primero?

1. **Comprensión y motivación (6:10 - 10 min.)**

Como familias embajadoras, su participación en la fase inicial del estudio ha sido muy importante para nosotros con el proyecto de Nuestra Comunidad Nuestra Salud. Sus comentarios en esta sesión nos ayudarán a mejorar nuestro diseño de estudio para que sea más beneficioso y útil para otras familias que están participando. Recuerde que puede usar el chat para responder a las preguntas, o para comentar que está de acuerdo con otros.

- 1. Para comenzar, ¿qué creen que este estudio está tratando de lograr? (Goals)
  2. ¿Qué les hizo querer participar en un estudio como este?
  3. ¿Por qué decidieron continuar?

1. **Experiencia (6:20 - 20 min.) (prioritize a, b, c, f)**
   1. ¿Cuál tarea fue la **más difícil** de completar? ¿Qué aspecto lo hizo difícil? ¿Por qué? ¿Qué lo haría más fácil?
   2. ¿Qué opinan sobre **la frecuencia (programación)** de las tareas de recopilación de datos?
   3. ¿Qué opinan sobre **el compromiso de tiempo** para cada una de las tareas?
      1. Optional probe: Al principio del estudio participaron en dos programas diferentes de recopilación de datos que se tomaron en el transcurso de unos meses. A partir del año siguiente les llevamos una carpeta del estudio con el nuevo cronograma. Con los cambios que hicimos, ¿Qué mejoró o empeoro el proceso?
   4. Después de participar en este estudio, ¿estaría interesado en participar en otros estudios con nosotros u otras organizaciones? ¿Por qué? ¿Por qué no?
   5. ¿Qué piensan sobre organizaciones de investigación como las universidades e instituciones gubernamentales? (Compare antes y después de su participación en el estudio, ¿qué cambió?)
   6. ¿Qué les ayudaría a confiar en una organización de investigación, y participar en estudios de investigación como este?
2. **Retroalimentación (6:40 - 5 min.)**
   1. Se le pidió retroalimentación sobre cómo se llevó a cabo el estudio, ¿cree que sus comentarios se tomaron en cuenta?
   2. ¿Cómo le gustaría recibir información sobre los resultados de este estudio? (por teléfono, en persona, llamada de Zoom, formato escrito por correo electrónico)
3. **Momentos de aprendizaje (6:45 - 10 min.)**
   1. ¿Qué aprendió sobre su familia al participar en esta investigación?
   2. ¿Qué parte de la recopilación de los datos fue la más útil e interesante para usted?
   3. Optional probe: Ha podido ver los cambios de la aplicación de Hornsense desde que comenzó la investigación. Al ver los cambios, ¿qué creen que ha mejorado y qué aspectos aún necesitan mejorar?
4. **Reflexión: Otras preguntas, ideas e historias de los participantes (6:55 - 5 min)**
   1. ¿Alguna pregunta o comentario que no hemos abordado?

**TURN OFF SCREEN SHARE**

1. **Conclusión**
   1. Gracias por tomar el tiempo para compartir sus pensamientos con nosotros. Sus comentarios nos ayudaran para mejorar el diseño de nuestro estudio. Actualmente estamos diseñando una plataforma para compartir los resultados del estudio con los participantes. En enero realizaremos entrevistas con cada familia embajadora (ustedes) para que compartan sus pensamientos y consejos en el proceso del diseño de la plataforma. Un miembro del equipo se comunicará con cada uno de ustedes para programar la entrevista, y cada participante será compensado con una tarjeta de regalo de $25.
   2. También quiero dejarles saber que la encuesta “retroalimentación del estudio” que nuestro equipo les compartió al principio del año pasado, pero que muchos de ustedes no podían ver, ya está disponible en la aplicación Hornsense. El problema está solucionado, y pueden llenar la encuesta en la aplicación del estudio. Solo les debe tomar 5 minutos. Nos sería muy útil ver cómo evalúa cada una de las tareas que completó durante el estudio. Recibirá una compensación de $5 al completar la encuesta.
   3. Por asistir el grupo hoy, recibirá una tarjeta de regalo de $25 mañana por correo electrónico.
   4. ¡Gracias!
