# Supplementary material for: Strategies to Implement a Community-Based, Longitudinal Cohort Study: The Whole Communities-Whole Health Case Study
Source: JMIR Form Res. 2024 Dec 5;8:e60368. doi: 10.2196/60368 (PMC11659690; doi:10.2196/60368)
Supplement: Multimedia Appendix 5 [file formative_v8i1e60368_app5.docx]

Ambassador Family Feedback Survey

[**Information Session**](#_b48a93ilakmv) **2**

[**Home Visit**](#_2gd4ikzfye5o) **3**

[**Surveys**](#_evm0dadhykj5) **4**

[**Dust samples**](#_8q0q9cc5bxdv) **5**

[**Saliva Samples**](#_kmlxra3liz0p) **6**

[**Accelerometer**](#_tdro3zdzqnr) **7**

[**LENA**](#_qlj8v2dangd8) **8**

[**Home sensor, Hornsense App, Fitbit**](#_ara2fckj2az6) **9**

# Information Session

1. Did you attend an information session virtually or in-person?

Virtual

In-person

2. How useful was the information that was covered at the information session?

Very useful

Somewhat useful

Not useful

3. How helpful were the resources included in the Welcome Folder?

Very helpful

Somewhat helpful

Not helpful

How could we make this experience more meaningful and relevant for the study participants? What do you suggest?

# Home Visit

1. Did you feel an hour was enough time for this visit to review the consent forms and complete the questionnaire?

It was enough time

It was not enough time

It was too much time

2. Did you feel you were provided with enough information about the study before joining?

Yes

No

Unsure

3. Did you find the resources you were sent based on the questionnaire, to be useful?

Very useful

Somewhat useful

Not helpful

4. Is there a different way you wish we would’ve sent this information?

Mail

Text

Other (please describe)

How could we make this experience more meaningful and relevant for the study participants? What do you suggest?

# Surveys

1. How easy was it to access and complete the surveys?

Very easy

Somewhat easy

Not easy

2. On average, how long did it take you to complete all surveys? (text box)

2a. Do you think there were too many surveys or it took too much time to complete them?

How could we make this experience more meaningful and relevant for the study participants? What do you suggest?

# Dust samples

1. How easy was it to collect the dust sample?

Very easy

Somewhat easy

Not easy

2. Were the instructions and information sent to you helpful in completing the sample?

Yes

No

Somewhat helpful but I wanted more assistance and/or information

How could we make this experience more meaningful and relevant for the study participants? What do you suggest?

# Saliva Samples

1. How easy was it to complete the saliva sample for you?

Very easy

Somewhat easy

Not easy

1a. How easy was it to complete for your child?

Very easy

Somewhat easy

Not easy

Not applicable

2. Were the instructions and information sent to you helpful in completing the sample?

Yes

No

Somewhat helpful but I wanted more assistance and/or information

How could we make this experience more meaningful and relevant for the study participants? What do you suggest?

# Accelerometer

1. How many days did your child agree to wear the “magic belt”?
2. How did the belt fit?

Too tight

Just right

Too loose and falling down

3. Did you read the books? If yes, did your child enjoy the book? If not, what would you recommend?

Yes

No

**If yes**, did your child enjoy the book?

**If no,** what would you recommend?

4. Was the daily wear log helpful for tracking the days your child wore the belt?

Yes

No (please describe)

How could we make this experience more meaningful and relevant for the study participants? What do you suggest?

# LENA

1. How easy was it to complete the LENA recording with your child?

Very easy

Somewhat easy

Not easy

2. Were the instructions and information sent to you helpful in completing the sample?

Yes

No

Somewhat helpful but I wanted more assistance and/or information

How could we make this experience more meaningful and relevant for the study participants? What do you suggest?

#

# Home sensor, Hornsense App, Fitbit

1. How easy was it to install the home sensor?

Very easy

Somewhat easy

Not easy

2. Were the instructions and information sent to you helpful in installing the home sensor?

Yes

No

Somewhat helpful but I wanted more assistance and/or information

3. Have you had other issues with the home sensor?

Please describe:

**Hornsense App**

1. How easy was it to install the mobile app?

Very easy

Somewhat easy

Not easy

2. How helpful has the app been in giving information about the study and helping you complete study related tasks?

Very helpful

Somewhat helpful

Not helpful

3. Have you had any issues with the mobile app so far?

If so, please describe:

**Fitbit**

1. How easy was it to set up your Fitbit?

Very easy

Somewhat easy

Not easy

2. Were the instructions and information sent to you helpful in setting up your Fitbit?

Yes

No

Somewhat helpful but I wanted more assistance and/or information

3. Have you had any issues with the FitBit?

Please describe:

4. Are you able to wear the Fitbit pretty regularly?

I wear it everyday

I wear it most days

I only wear it sometimes but not often

If most days or not often -

Are there any issues that keep you from wearing it? Please describe:

How could we make this experience more meaningful and relevant for the study participants? What do you suggest?

# Water samples

1. How easy was it to collect the water samples?

Very easy

Somewhat easy

Not easy

2. Were the instructions and information sent to you helpful?

Yes

No

Any suggestions on how the instructions could be more clear?

3. Would you be willing to do the water samples twice a year the same way? (2 times a week for 4 weeks)

4. Instead of collecting water samples 2 times a week for 4 weeks, would you be willing to collect 1 sample per day for 1 week?

How could we make this experience more meaningful and relevant for the study participants? What do you suggest?

Communication with the research team

1. How easy was it to get in touch with us if you needed something or had a question?
   1. Very easy
   2. Somewhat easy
   3. Not easy
2. Did the way we communicated with you about scheduling, study material drop-offs/pick-ups, and general information work for you?
   1. Yes
   2. No
   3. Is there another way to reach you that you would prefer we use more often?
